# The genome sequence of the Common Quaker, *Orthosia cerasi* (Fabricius, 1775) (Lepidoptera: Noctuidae)

**DOI:** 10.12688/wellcomeopenres.26626.1

**Published:** 2026-05-21

**Authors:** David C. Lees, Nick Crumpton, Natural History Museum Genome Acquisition Lab, Darwin Tree of Life Barcoding Collective, Wellcome Sanger Institute Tree of Life Management, Samples and Laboratory team, Wellcome Sanger Institute Scientific Operations: Sequencing Operations, Wellcome Sanger Institute Tree of Life Core Informatics team, Tree of Life Core Informatics collective, Darwin Tree of Life Consortium

**Affiliations:** 1 Natural History Museum, London, England, UK

**Keywords:** Orthosia cerasi, Common Quaker, genome sequence, chromosomal, Lepidoptera

## Abstract

We present a genome assembly from an individual female *Orthosia cerasi* (Common Quaker; Arthropoda; Insecta; Lepidoptera; Noctuidae). The genome sequence has a total length of 1 022.21 megabases. Most of the assembly (99.88%) is scaffolded into 29 chromosomal pseudomolecules, including the Z sex chromosome. The mitochondrial genome has also been assembled, with a length of 15.39 kilobases. Gene annotation of this assembly on Ensembl identified 15 702 protein-coding genes. This assembly was generated as part of the Darwin Tree of Life project, which produces genomes for eukaryotic species found in Britain and Ireland.

## Species taxonomy

Eukaryota; Metazoa; Arthropoda; Insecta; Lepidoptera; Noctuidae; *Orthosia*; *Orthosia cerasi* (Fabricius, 1775) (NCBI:txid875886).

## Background

*Orthosia cerasi* (Fabricius, 1775) is a hadenine noctuid moth, the most common of the “Quakers”. This medium-sized, nocturnal species has a wingspan of 34–40 mm, and bipectinate antennae in the male. It is readily attracted to light and can be recognised by its round-tipped brownish to greyish wings and kidney-shaped stigmata ( Heath & Emmet, 1983). The species is most abundant at low elevations and in woodlands, though also common in more open habitats such as suburban gardens; its larva feeds on a wide variety of broad-leaved trees including oak, elm, hawthorn and birch ( Waring *et al.*, 2017).

*Orthosia* species are predominantly univoltine, with *O. cerasi* caterpillars emerging from green-grey eggs about 10 days after laying. Caterpillars are ~38 mm long when fully fed, green, large-headed, and spotted with black dots and pale stripes the length of the body ( Heath & Emmet, 1983). Adults emerge from subterranean cocoons from March to May, while larvae are active from April to June ( Waring *et al.*, 2017) in the British Isles.

Common Quaker *O. cerasi* occurs widely throughout Britain as far north as the Orkney Islands. It is widespread and common in Ireland, and populations have been growing in the south-west since the 1970s ( Randle *et al.*, 2019). This species also occurs widely in Europe and western Asia (e.g. Poorshabanan & Shirvani, 2022), and has been documented as far east as central Russia ( GBIF Secretariat, 2023). Although *O. cerasi* is historically widespread in the UK, recent populations appear to be reducing, a decline which appears statistically linked to warming temperatures ( Bell *et al.*, 2020) and resultant ecological issues including vegetation-larval developmental mismatch ( Senior *et al.*, 2021). Furthermore, the development of *O. cerasi* has been shown experimentally to be sensitive to both light availability and temperature, with faster larval growth in environments with increased ambient temperatures ( Reinhard *et al.*, 2026). Although growth was negatively impacted by artificially increased light levels, these results indicate that the development of macromoths such as *O. cerasi* may be subtly impacted by climate change.

The DNA barcode from the mitochondrial assembly (OZ217469.1) belongs to BOLD cluster BOLD:AAC3426 (accessed 2026-04-27). The closest species-level match on BOLD is *Orthosia dalmatica* (Wagner, 1909), represented by BGE_00150_D04, which differs from *O. cerasi* by a *p*-distance of approximately 3.06%. *Orthosia dalmatica* is distributed in Greece and Asia Minor.

**Figure 1.**
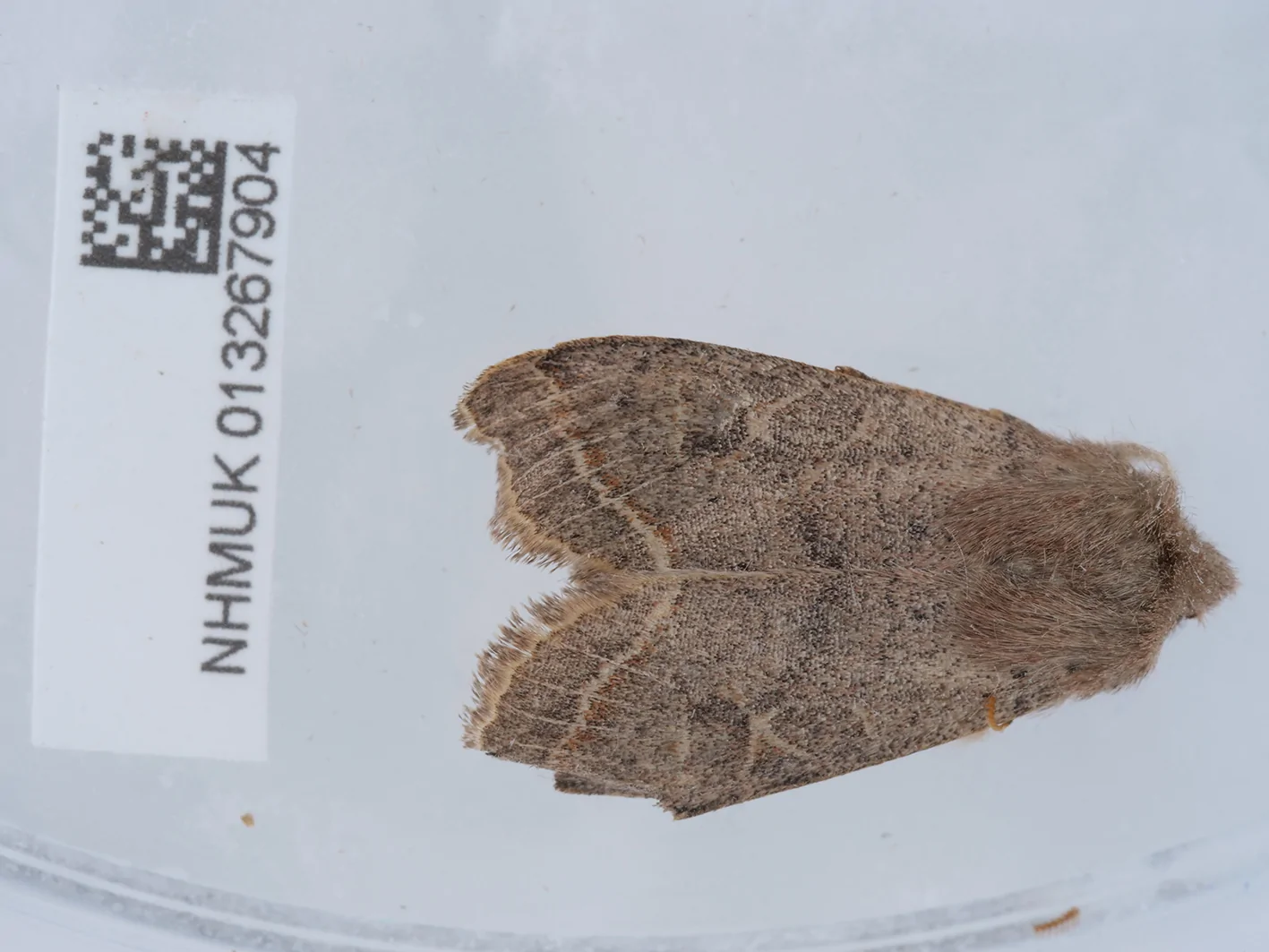
Photograph of the *Orthosia cerasi* (ilOrtCera1) specimen used for genome sequencing.

We present the first chromosome-level genome sequence for *Orthosia cerasi*, produced by the Tree of Life pipeline from a specimen collected in High Wycombe, UK ( Figure 1).

## Methods

### Sample acquisition and DNA barcoding

The specimen used for genome sequencing was an adult female *Orthosia cerasi* (specimen ID NHMUK013267904, ToLID ilOrtCera1; Figure 1), collected from Lucas Road, High Wycombe, Buckinghamshire, UK (latitude 51.63, longitude −0.74) on 2021-02-16. The specimen was collected and identified by David Lees. The same specimen was used for RNA sequencing.

The initial identification was verified by an additional DNA barcoding process according to the framework developed by Twyford *et al.* (2024). A small sample was dissected from the specimen and stored in ethanol, while the remaining parts were shipped on dry ice to the Wellcome Sanger Institute (WSI) (see the protocol). The tissue was lysed, the COI marker region was amplified by PCR, and amplicons were sequenced and compared to the BOLD database, confirming the species identification ( Crowley *et al.*, 2023). Following whole genome sequence generation, the relevant DNA barcode region was also used alongside the initial barcoding data for sample tracking at the WSI ( Twyford *et al.*, 2024). The standard operating procedures for Darwin Tree of Life barcoding are available on protocols.io.

### Nucleic acid extraction

Detailed protocols for nucleic acid extraction developed at the Wellcome Sanger Institute (WSI) Tree of Life Core Laboratory are available on protocols.io ( Howard *et al.*, 2025). The ilOrtCera1 sample was weighed and triaged to determine the appropriate extraction protocol. For HMW DNA extraction, tissue from the head and thorax was used. Tissue was homogenised by powermashing using a PowerMasher II tissue disruptor. High molecular weight (HMW) DNA was extracted using the Automated MagAttract v2 protocol. We used centrifuge-mediated fragmentation to produce DNA fragments in the 8–10 kb range, following the Covaris g-TUBE protocol for ultra-low input (ULI). Sheared DNA was purified by automated SPRI (solid-phase reversible immobilisation). The concentration of the sheared and purified DNA was assessed using a Nanodrop spectrophotometer and Qubit Fluorometer using the Qubit dsDNA High Sensitivity Assay kit. Fragment size distribution was evaluated by running the sample on the.

RNA was extracted from abdomen tissue of ilOrtCera1 in the Tree of Life Laboratory at the WSI using the RNA Extraction: Automated MagMax™ *mir*Vana protocol. The RNA concentration was assessed using a Nanodrop spectrophotometer and a Qubit Fluorometer using the Qubit RNA Broad-Range Assay kit. Analysis of the integrity of the RNA was done using the Agilent RNA 6000 Pico Kit and Eukaryotic Total RNA assay.

### PacBio HiFi library preparation and sequencing

Library preparation and sequencing were performed at the WSI Scientific Operations core. Prior to library preparation, the DNA was fragmented to ~10 kb. Ultra-low-input (ULI) libraries were prepared using the PacBio SMRTbell® Express Template Prep Kit 2.0 and gDNA Sample Amplification Kit. Samples were normalised to 20 ng DNA. Single-strand overhang removal, DNA damage repair, and end-repair/A-tailing were performed according to the manufacturer’s instructions, followed by adapter ligation. A 0.85× pre-PCR clean-up was carried out with Promega ProNex beads.

The DNA was evenly divided into two aliquots for dual PCR (reactions A and B), both following the manufacturer’s protocol. A 0.85× post-PCR clean-up was performed with ProNex beads. DNA concentration was measured using a Qubit Fluorometer v4.0 (Thermo Fisher Scientific) with the Qubit HS Assay Kit, and fragment size was assessed on an Agilent Femto Pulse Automated Pulsed Field CE Instrument (Agilent Technologies) using the gDNA 55 kb BAC analysis kit. PCR reactions A and B were then pooled, ensuring a total mass of ≥500 ng in 47.4 μl.

The pooled sample underwent another round of DNA damage repair, end-repair/A-tailing, and hairpin adapter ligation. A 1× clean-up was performed with ProNex beads, followed by DNA quantification using the Qubit and fragment size analysis using the Agilent Femto Pulse. Size selection was performed on the Sage Sciences PippinHT system, with target fragment size determined by Femto Pulse analysis (typically 4–9 kb). Size-selected libraries were cleaned with 1.0× ProNex beads and normalised to 2 nM before sequencing.

The sample was sequenced using the Sequel IIe system (Pacific Biosciences, California, USA). The concentration of the library loaded onto the Sequel IIe was in the range 40–135 pM. The SMRT link software, a PacBio web-based end-to-end workflow manager, was used to set-up and monitor the run, and to perform primary and secondary analysis of the data upon completion.

### Hi-C

***Sample preparation and crosslinking***

The Hi-C sample was prepared from 20–50 mg of frozen head and thorax tissue of the ilOrtCera1 sample using the Arima-HiC v2 kit (Arima Genomics). Following the manufacturer’s instructions, tissue was fixed and DNA crosslinked using TC buffer to a final formaldehyde concentration of 2%. The tissue was homogenised using the Diagnocine Power Masher-II. Crosslinked DNA was digested with a restriction enzyme master mix, biotinylated, and ligated. Clean-up was performed with SPRISelect beads before library preparation. DNA concentration was measured with the Qubit Fluorometer (Thermo Fisher Scientific) and Qubit HS Assay Kit. The biotinylation percentage was estimated using the Arima-HiC v2 QC beads.

***Hi-C library preparation and sequencing***

Biotinylated DNA constructs were fragmented using a Covaris E220 sonicator and size selected to 400–600 bp using SPRISelect beads. DNA was enriched with Arima-HiC v2 kit Enrichment beads. End repair, A-tailing, and adapter ligation were carried out with the NEBNext Ultra II DNA Library Prep Kit (New England Biolabs), following a modified protocol where library preparation occurs while DNA remains bound to the Enrichment beads. Library amplification was performed using KAPA HiFi HotStart mix and a custom Unique Dual Index (UDI) barcode set (Integrated DNA Technologies). Depending on sample concentration and biotinylation percentage determined at the crosslinking stage, libraries were amplified with 10–16 PCR cycles. Post-PCR clean-up was performed with SPRISelect beads. Libraries were quantified using the AccuClear Ultra High Sensitivity dsDNA Standards Assay Kit (Biotium) and a FLUOstar Omega plate reader (BMG Labtech).

Prior to sequencing, libraries were normalised to 10 ng/μL. Normalised libraries were quantified again to create equimolar and/or weighted 2.8 nM pools. Pool concentrations were checked using the Agilent 4200 TapeStation (Agilent) with High Sensitivity D500 reagents before sequencing. Sequencing was performed using paired-end 150 bp reads on the Illumina NovaSeq 6000.

### RNA library preparation and sequencing

Libraries were prepared using the NEBNext® Ultra™ II Directional RNA Library Prep Kit for Illumina (New England Biolabs), following the manufacturer’s instructions. Poly(A) mRNA in the total RNA solution was isolated using oligo (dT) beads, converted to cDNA, and uniquely indexed; 14 PCR cycles were performed. Libraries were size-selected to produce fragments between 100–300 bp. Libraries were quantified, normalised, pooled to a final concentration of 2.8 nM, and diluted to 150 pM for loading. Sequencing was carried out on the Illumina NovaSeq 6000, generating paired-end reads.

### Genome assembly

Prior to assembly of the PacBio HiFi reads, a database of *k*-mer counts ( *k* = 31) was generated from the filtered reads using FastK. GenomeScope2 ( Ranallo-Benavidez *et al.*, 2020) was used to analyse the *k*-mer frequency distributions, providing estimates of genome size, heterozygosity, and repeat content.

The HiFi reads were assembled using Hifiasm ( Cheng *et al.*, 2021) with the --primary option. Haplotypic duplications were identified and removed using purge_dups ( Guan *et al.*, 2020). The Hi-C reads ( Rao *et al.*, 2014) were mapped to the primary contigs using bwa-mem2 ( Vasimuddin *et al.*, 2019), and the contigs were scaffolded in YaHS ( Zhou *et al.*, 2023) with the --break option for handling potential misassemblies. The scaffolded assemblies were evaluated using Gfastats ( Formenti *et al.*, 2022), BUSCO ( Manni *et al.*, 2021) and MerquryFK ( Rhie *et al.*, 2020).

The mitochondrial genome was assembled using MitoHiFi ( Uliano-Silva *et al.*, 2023).

### Assembly curation

The assembly was decontaminated using the Assembly Screen for Cobionts and Contaminants ( ASCC) pipeline. TreeVal was used to generate the flat files and maps for use in curation. Manual curation was conducted primarily in PretextView and HiGlass ( Kerpedjiev *et al.*, 2018). Scaffolds were visually inspected and corrected as described by Howe *et al*. (2021). Manual corrections included 258 breaks and 530 joins. This reduced the scaffold count by 9.7%, reduced the scaffold N50 by 2.6%, and reduced the total assembly length by 2.7%. The curation process is described at https://gitlab.com/wtsi-grit/rapid-curation . PretextSnapshot was used to generate a Hi-C contact map of the final assembly.

### Assembly quality assessment

The MerquryFK tool ( Rhie *et al.*, 2020) was run in a Singularity container ( Kurtzer *et al.*, 2017) to evaluate *k*-mer completeness and assembly quality for the primary and alternate haplotypes using the *k*-mer database ( *k* = 31) computed prior to genome assembly. The analysis outputs included assembly QV scores and completeness statistics.

The genome was analysed using the BlobToolKit pipeline, a Nextflow implementation of the earlier Snakemake version ( Challis *et al.*, 2020). The pipeline aligns PacBio reads using minimap2 ( Li, 2018) and SAMtools ( Danecek *et al.*, 2021) to generate coverage tracks. It runs BUSCO ( Manni *et al.*, 2021) using lineages identified from the NCBI Taxonomy ( Schoch *et al.*, 2020). For the three domain-level lineages, BUSCO genes are aligned to the UniProt Reference Proteomes database ( Bateman *et al.*, 2023) using DIAMOND blastp ( Buchfink *et al.*, 2021). The genome is divided into chunks based on the density of BUSCO genes from the closest taxonomic lineage, and each chunk is aligned to the UniProt Reference Proteomes database with DIAMOND blastx. Sequences without hits are chunked using seqtk and aligned to the NT database with blastn ( Altschul *et al.*, 1990). The BlobToolKit suite consolidates all outputs into a blobdir for visualisation. The BlobToolKit pipeline was developed using nf-core tooling ( Ewels *et al.*, 2020) and MultiQC ( Ewels *et al.*, 2016), with containerisation through Docker ( Merkel, 2014) and Singularity ( Kurtzer *et al.*, 2017).

## Genome sequence report

### Sequence data

**Figure 2.**
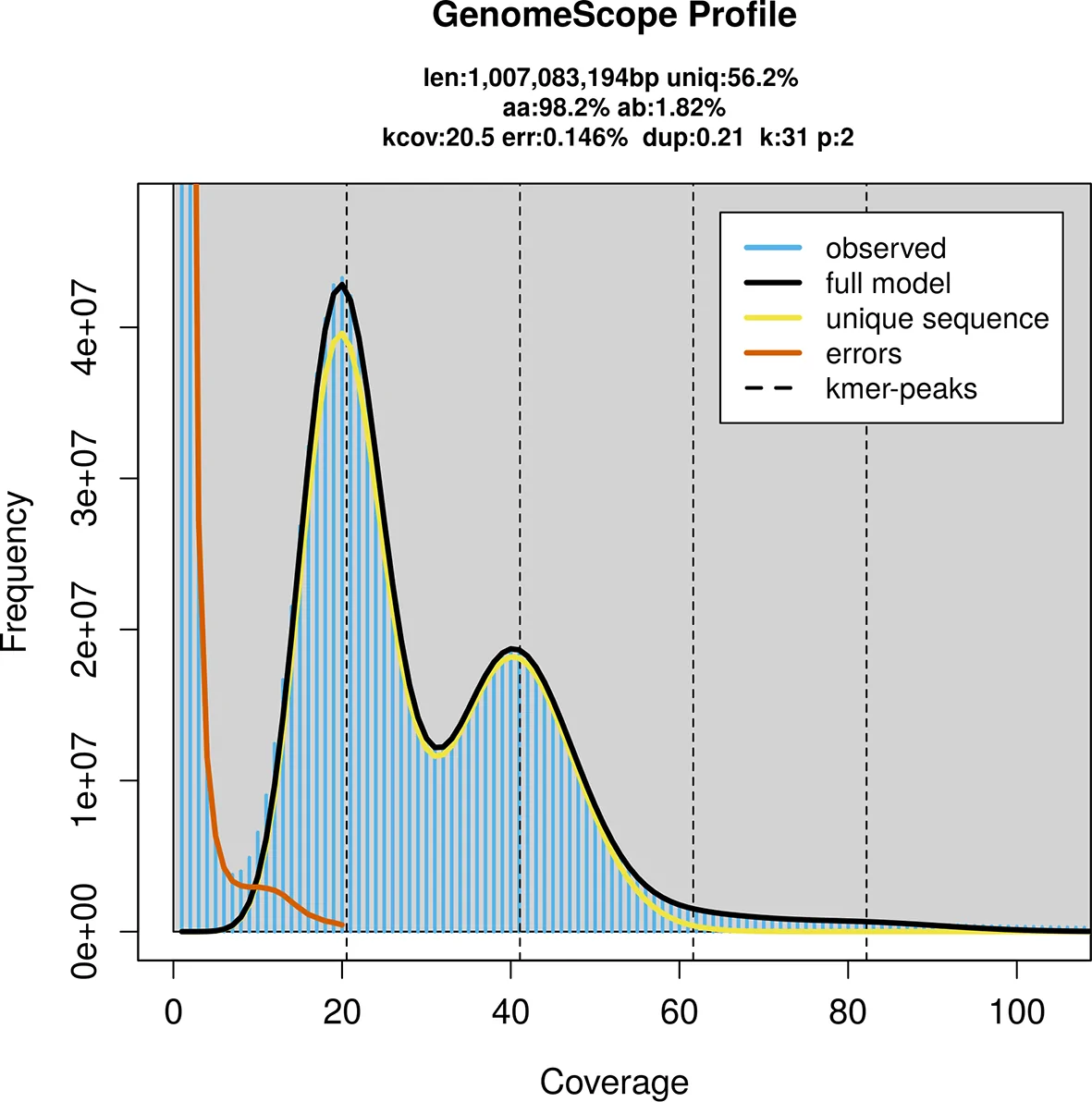
Frequency distribution of *k*-mers generated using GenomeScope2. The plot shows observed and modelled *k*-mer spectra, providing estimates of genome size, heterozygosity, and repeat content based on unassembled sequencing reads.

PacBio sequencing of the *Orthosia cerasi* specimen generated 44.54 Gb (gigabases) from 4.61 million reads, which were used to assemble the genome. GenomeScope2.0 analysis estimated the haploid genome size at 1 007.08 Mb, with a heterozygosity of 1.82% and repeat content of 43.99% ( Figure 2). These estimates guided expectations for the assembly. Based on the estimated genome size, the sequencing data provided approximately 41× coverage. Hi-C sequencing produced 114.13 Gb from 377.93 million reads, which were used to scaffold the assembly. RNA sequencing data were also generated and are available in public sequence repositories. Table 1 summarises the specimen and sequencing details.

**Table 1.** Specimen and sequencing data for *Orthosia cerasi* (BioProject PRJEB56259).

Platform	PacBio HiFi	Hi-C	RNA-seq
**ToLID**	ilOrtCera1	ilOrtCera1	ilOrtCera1
**Specimen ID**	NHMUK013267904	NHMUK013267904	NHMUK013267904
**BioSample (source individual)**	SAMEA9359454	SAMEA9359454	SAMEA9359454
**BioSample (tissue)**	SAMEA9359547	SAMEA9359547	SAMEA9359548
**Tissue**	head and thorax	head and thorax	abdomen
**Instrument**	Sequel II	Illumina NovaSeq 6000	Illumina NovaSeq 6000
**Run accessions**	ERR10355978; ERR10355977	ERR10297881	ERR10908609
**Read count total**	4.61 million	377.93 million	32.94 million
**Base count total**	44.54 Gb	114.13 Gb	9.95 Gb

### Assembly statistics

The primary haplotype was assembled, and contigs corresponding to an alternate haplotype were also deposited in INSDC databases. The final assembly has a total length of 1 022.21 Mb in 55 scaffolds, with 564 gaps, and a scaffold N50 of 35.9 Mb ( Table 2).

**Table 2.** Genome assembly data for *Orthosia cerasi.*

Genome assembly	Primary assembly
**Assembly name**	ilOrtCera1.1
**Assembly accession**	GCA_965111875.1
**Alternate haplotype accession**	GCA_965111865.1
**Assembly level**	chromosome
**Span (Mb)**	1 022.21
**Number of chromosomes**	29
**Number of contigs**	619
**Contig N50**	4.0 Mb
**Number of scaffolds**	55
**Scaffold N50**	35.9 Mb
**Sex chromosomes**	Z
**Organelles**	Mitochondrion: 15.39 kb

**Figure 3.**
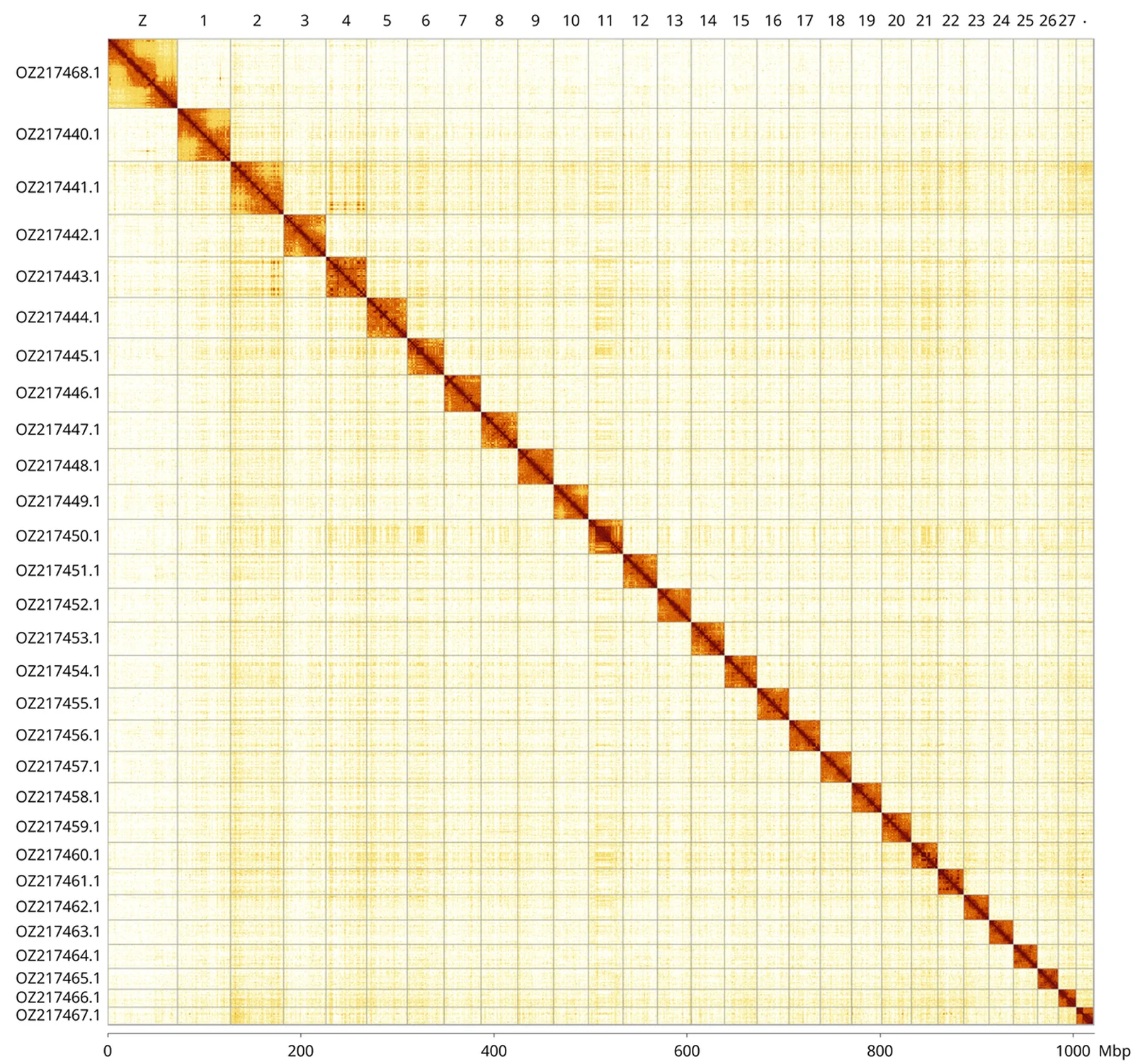
Hi-C contact map of the *Orthosia cerasi* genome assembly. Assembled chromosomes are shown in order of size and labelled along the axes, with a megabase scale shown below. The plot was generated using PretextSnapshot.

Most of the assembly sequence (99.88%) was assigned to 29 chromosomal-level scaffolds, representing 28 autosomes and the Z sex chromosome. These chromosome-level scaffolds, confirmed by Hi-C data, are named according to size ( Figure 3; Table 3). Chromosome Z was identified by BUSCO gene painting with ancestral Merian elements ( Wright *et al.*, 2024).

**Table 3.** Chromosomal pseudomolecules in the primary genome assembly of *Orthosia cerasi* ilOrtCera1.

INSDC accession	Molecule	Length (Mb)	GC%
OZ217440.1	1	55.06	38.50
OZ217441.1	2	54.99	38.50
OZ217442.1	3	43.92	38.50
OZ217443.1	4	42.13	38.50
OZ217444.1	5	41.95	38
OZ217445.1	6	38.30	38.50
OZ217446.1	7	38.16	38.50
OZ217447.1	8	37.91	38
OZ217448.1	9	37.11	38.50
OZ217449.1	10	36.26	38.50
OZ217450.1	11	35.90	38
OZ217451.1	12	35.57	38.50
OZ217452.1	13	34.89	38.50
OZ217453.1	14	34.58	38
OZ217454.1	15	33.56	38.50
OZ217455.1	16	33.35	38
OZ217456.1	17	32.39	39
OZ217457.1	18	32.39	38.50
OZ217458.1	19	31.02	38.50
OZ217459.1	20	30.71	38.50
OZ217460.1	21	27.54	38.50
OZ217461.1	22	26.79	38.50
OZ217462.1	23	26.27	38.50
OZ217463.1	24	25.14	38
OZ217464.1	25	24.96	38
OZ217465.1	26	21.50	38.50
OZ217466.1	27	18.63	39
OZ217467.1	28	17.80	40
OZ217468.1	Z	72.25	38

The mitochondrial genome was also assembled (length 15.39 kb, OZ217469.1). This sequence is included as a contig in the multifasta file of the genome submission and as a standalone record.

### Assembly quality metrics

**Figure 4.**
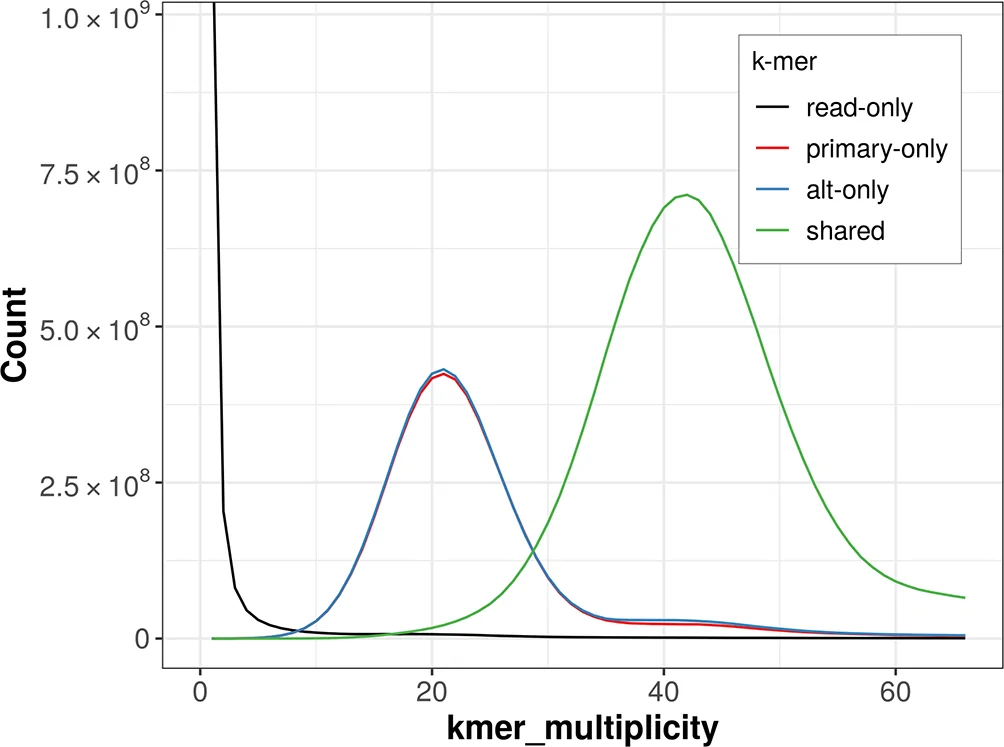
Evaluation of *k*-mer completeness using MerquryFK. This plot illustrates the recovery of *k*-mers from the original read data in the final assemblies. The horizontal axis represents *k*-mer multiplicity, and the vertical axis shows the number of *k*-mers. The black curve represents *k*-mers that appear in the reads but are not assembled. The green curve corresponds to *k*-mers shared by both haplotypes, and the red and blue curves show *k*-mers found only in one of the haplotypes.

The combined primary and alternate assemblies achieve an estimated QV of 63.6. The *k*-mer completeness is 69.28% for the primary assembly, 69.84% for the alternate haplotype, and 98.74% for the combined assemblies ( Figure 4).

**Figure 5.**
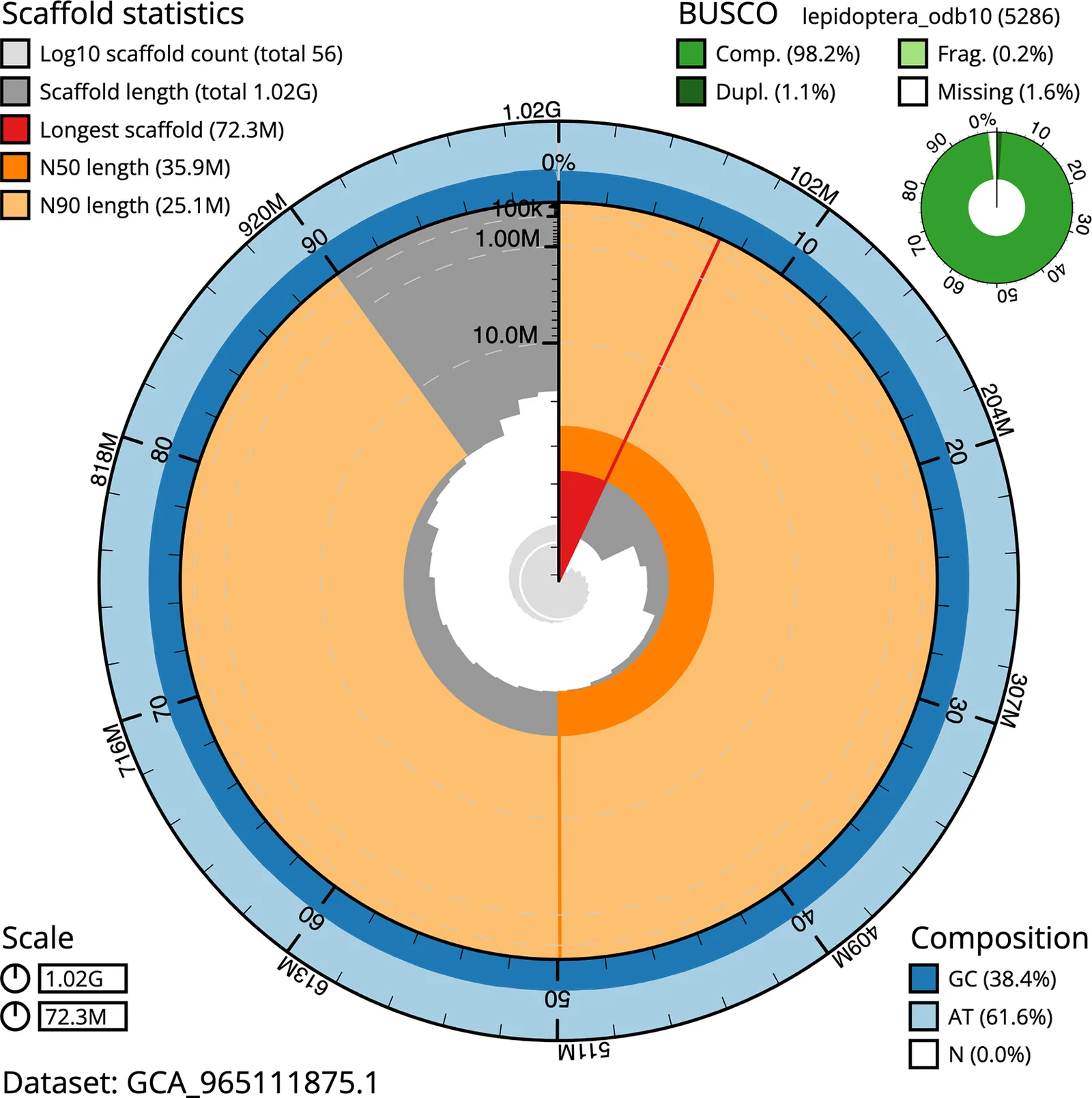
Assembly metrics for ilOrtCera1.1. The BlobToolKit snail plot provides an overview of assembly metrics and BUSCO gene completeness. The circumference represents the length of the whole genome sequence, and the main plot is divided into 1 000 bins around the circumference. The outermost blue tracks display the distribution of GC, AT, and N percentages across the bins. Scaffolds are arranged clockwise from longest to shortest and are depicted in dark grey. The longest scaffold is indicated by the red arc, and the deeper orange and pale orange arcs represent the N50 and N90 lengths. A light grey spiral at the centre shows the cumulative scaffold count on a logarithmic scale. A summary of complete, fragmented, duplicated, and missing BUSCO genes in the lepidoptera_odb10 set is presented at the top right. An interactive version of this figure can be accessed on the BlobToolKit viewer.

**Figure 6.**
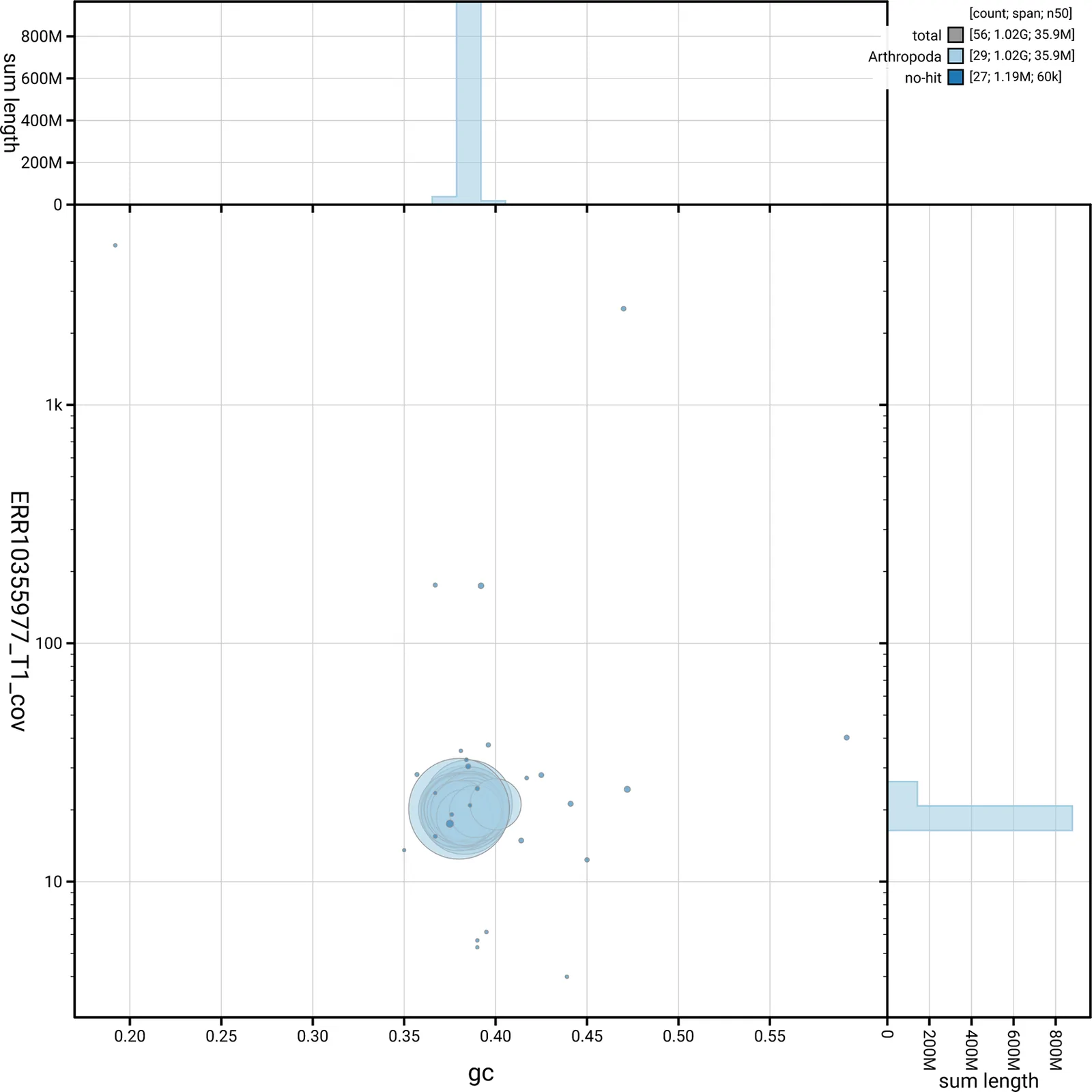
BlobToolKit blob plot for ilOrtCera1.1. The plot shows base coverage (vertical axis) and GC content (horizontal axis). The circles represent scaffolds, with the size proportional to scaffold length and the colour representing phylum membership. The histograms along the axes display the total length of sequences distributed across different levels of coverage and GC content. An interactive version of this figure is available on the BlobToolKit viewer.

BUSCO v.5.5.0 analysis using the lepidoptera_odb10 reference set ( *n* = 5 286) identified 98.2% of the expected gene set (single = 97.0%, duplicated = 1.1%). The snail plot in Figure 5 summarises the scaffold length distribution and other assembly statistics for the primary assembly. The blob plot in Figure 6 shows the distribution of scaffolds by GC proportion and coverage.

Table 4 lists the assembly metric benchmarks adapted from Rhie *et al.* (2021) and the Earth BioGenome Project Report on Assembly Standards. The EBP metric, calculated for the primary assembly, is **6.C.Q63**, meeting the recommended reference standard.

**Table 4.** Earth biogenome project summary metrics for the *Orthosia cerasi* assembly.

Measure	Value	Benchmark
EBP summary (primary)	6.C.Q63	6.C.Q40
Contig N50 length	4 Mb	≥ 1 Mb
Scaffold N50 length	35.90 Mb	= chromosome N50
Consensus quality (QV)	Primary: 63.9; alternate: 63.4; combined: 63.6	≥ 40
*k*-mer completeness	Primary: 69.28%; alternate: 69.84%; combined: 98.74%	≥ 95%
BUSCO	C:98.2% [S:97.0%, D:1.1%], F:0.2%, M:1.6%, n:5 286	S > 90%; D < 5%
Percentage of assembly assigned to chromosomes	99.88%	≥ 90%

**Notes:** The EBP summary uses log10(Contig N50); chromosome-level (C) or log10(Scaffold N50); Q (Merqury QV). BUSCO: C = complete; S = single-copy; D = duplicated; F = fragmented; M = missing; n = orthologues

**Table 5.** Software versions and sources used for *Orthosia cerasi.*

Software	Version	Source
BLAST	2.14.0	ftp://ftp.ncbi.nlm.nih.gov/blast/executables/blast+/
BlobToolKit	4.3.9	https://github.com/blobtoolkit/blobtoolkit
BUSCO	5.5.0	https://gitlab.com/ezlab/busco
bwa-mem2	2.2.1	https://github.com/bwa-mem2/bwa-mem2
DIAMOND	2.1.8	https://github.com/bbuchfink/diamond
fasta_windows	0.2.4	https://github.com/tolkit/fasta_windows
FastK	1.1	https://github.com/thegenemyers/FASTK
GenomeScope2.0	2.0.1	https://github.com/tbenavi1/genomescope2.0
Gfastats	1.3.6	https://github.com/vgl-hub/gfastats
Hifiasm	0.19.5-r587	https://github.com/chhylp123/hifiasm
HiGlass	1.13.4	https://github.com/higlass/higlass
MerquryFK	1.1.2	https://github.com/thegenemyers/MERQURY.FK
Minimap2	2.24-r1122	https://github.com/lh3/minimap2
MitoHiFi	3	https://github.com/marcelauliano/MitoHiFi
MultiQC	1.14; 1.17 and 1.18	https://github.com/MultiQC/MultiQC
Nextflow	23.10.0	https://github.com/nextflow-io/nextflow
PretextSnapshot	0.0.5	https://github.com/sanger-tol/PretextSnapshot
PretextView	1.0.3	https://github.com/sanger-tol/PretextView
purge_dups	1.2.5	https://github.com/dfguan/purge_dups
samtools	1.19.2	https://github.com/samtools/samtools
sanger-tol/ascc	0.1.0	https://github.com/sanger-tol/ascc
sanger-tol/blobtoolkit	0.6.0	https://github.com/sanger-tol/blobtoolkit
sanger-tol/curationpretext	1.4.2	https://github.com/sanger-tol/curationpretext
Seqtk	1.3	https://github.com/lh3/seqtk
Singularity	3.9.0	https://github.com/sylabs/singularity
TreeVal	1.4.0	https://github.com/sanger-tol/treeval
YaHS	1.2a.2	https://github.com/c-zhou/yahs

### Genome annotation report

The *Orthosia cerasi* genome assembly (GCA_965111875.1) was annotated by Ensembl at the European Bioinformatics Institute (EBI). This annotation includes 32 539 transcribed mRNAs from 15 702 protein-coding and 4 616 non-coding genes. The average transcript length is 21 001.77 bp, with an average of 1.60 coding transcripts per gene and 6.83 exons per transcript. Further details of this annotation are available from the Ensembl annotation page.

## Author information

Contributors are listed at the following links:

• Members of the Natural History Museum Genome Acquisition Lab
• Members of the Darwin Tree of Life Barcoding collective
• Members of the Wellcome Sanger Institute Tree of Life Management, Samples and Laboratory team
• Members of Wellcome Sanger Institute Scientific Operations – Sequencing Operations
• Members of the Wellcome Sanger Institute Tree of Life Core Informatics team
• Members of the Tree of Life Core Informatics collective
• Members of the Darwin Tree of Life Consortium

## Wellcome sanger institute – Legal and governance

The materials that have contributed to this genome note have been supplied by a Darwin Tree of Life Partner. The submission of materials by a Darwin Tree of Life Partner is subject to the **‘Darwin Tree of Life Project Sampling Code of Practice’**, which can be found in full on the Darwin Tree of Life website. By agreeing with and signing up to the Sampling Code of Practice, the Darwin Tree of Life Partner agrees they will meet the legal and ethical requirements and standards set out within this document in respect of all samples acquired for, and supplied to, the Darwin Tree of Life Project. Further, the Wellcome Sanger Institute employs a process whereby due diligence is carried out proportionate to the nature of the materials themselves, and the circumstances under which they have been/are to be collected and provided for use. The purpose of this is to address and mitigate any potential legal and/or ethical implications of receipt and use of the materials as part of the research project, and to ensure that in doing so we align with best practice wherever possible. The overarching areas of consideration are:

• Ethical review of provenance and sourcing of the material
• Legality of collection, transfer and use (national and international)

Each transfer of samples is further undertaken according to a Research Collaboration Agreement or Material Transfer Agreement entered into by the Darwin Tree of Life Partner, Genome Research Limited (operating as the Wellcome Sanger Institute), and in some circumstances, other Darwin Tree of Life collaborators.

## Data Availability

European Nucleotide Archive: Orthosia cerasi. Accession number PRJEB56259. The genome sequence is released openly for reuse. The *Orthosia cerasi* genome sequencing initiative is part of the Darwin Tree of Life Project (PRJEB40665), Sanger Institute Tree of Life Programme (PRJEB43745) and Project Psyche (PRJEB71705). All raw sequence data and the assembly have been deposited in INSDC databases. Raw data and assembly accession identifiers are reported in Tables 1 and 2. Production code used in genome assembly at the WSI Tree of Life is available at https://github.com/sanger-tol . Table 5 lists software versions used in this study.
