# Supplementary material for: The genome sequence of the Common Quaker, Orthosia cerasi (Fabricius, 1775) (Lepidoptera: Noctuidae)
Source: Wellcome Open Res. 2026 May 21;11:294. [Version 1] doi: 10.12688/wellcomeopenres.26626.1 (PMC13555078; doi:10.12688/wellcomeopenres.26626.1)
Supplement: Peer review history

# Reviewer report: Reviewer response for version 1

**DOI:** 10.21956/wellcomeopenres.29322.r161845

**Published:** 2026-08-10

**Authors:** Jayan Duminda M Senevirathna

**Affiliations:** 1 Uva Wellassa University, Badulla, Uva Province, Sri Lanka

Scientific names used in the keywords should be italicized in accordance with standard scientific nomenclature.

 RNA was extracted from abdominal tissue. The authors should clarify whether the use of this particular tissue could introduce tissue-specific selection or bias in the genes identified and analyzed. Please provide a justification for selecting the abdomen as the RNA source and discuss any potential limitations.

 A Z chromosome has been identified in this insect. The authors should clarify the sex-determination system of the species, particularly whether it follows a ZZ/ZW system, with females being heterogametic (ZW). If females are ZW, it is important to explain whether the W chromosome was identified and/or assembled in the current genome assembly. If the W chromosome was not assembled, the authors should provide the reason and discuss the potential implications for the completeness and interpretation of the genome assembly.

Are sufficient details of methods and materials provided to allow replication by others?

Yes

Is the rationale for creating the dataset(s) clearly described?

Yes

Are the datasets clearly presented in a useable and accessible format?

Yes

Are the protocols appropriate and is the work technically sound?

Yes

Reviewer Expertise:

Genomics and biotechnology

I confirm that I have read this submission and believe that I have an appropriate level of expertise to confirm that it is of an acceptable scientific standard, however I have significant reservations, as outlined above.

# Reviewer report: Reviewer response for version 1

**DOI:** 10.21956/wellcomeopenres.29322.r156294

**Published:** 2026-07-25

**Authors:** Jurate De Prins

**Affiliations:** 1 Belgian Institute of Natural Sciences, Brussels, Belgium; 2 Flemish Entomological Society, Vrieselhof, Belgium

In general, the articles of the Wellcome Sanger Institute are methodological and report the whole genome of an insect species. I believe that, regarding molecular aspects, everything is done that modern technology allows to present. The authors' attention probably needs to be paid to details in presenting taxonomic, ecological and karyological data.

**Taxonomy**

 As I mentioned in my former reviews, the correct species identification is crucial for further whole-genome analysis. In Belgium, just across the Channel, 8 species of *Orthosia* occur, 7 of which are very common; please see https://projects.biodiversity.be/lepidoptera/genus/1332/.  It is very good that the authors checked the ID against the DNA barcodes, but whether they checked against all 8 *Orthosia* species? What is the difference? If it is less than 100%, then it needs an explanation since sometimes our Noctuidae entomologists describe species with tiny DNA barcode differences. We all remember the great discussion 15 years ago when the Noctuidae family was split based solely on the DNA. 

*Orthosia stabilis* (Denis & Schiffermüller, 1775) is a synonym of *O. cerasi*.  Is the DNA barcode and the whole genome also the same?

**Ecology**

 In Belgium, *O. cerasi* was observed only on *Quercus* and *Salix,* while the authors suggest that larvae feed on oak, elm, hawthorn, and birch. I would recommend adding Latin names for plants also, and if possible, to be more precise about host plants.

**Karyology**

 Since the sample was a female, so the sex chromosome system is heterogametic. The chromosome set is presented in the article. It is very important to inform the readers whether the sex chromosome system of this species is Z0 or WZ. Keeping in mind that 29 chromosomes are present in this species and not 30 as one can expect in higher moths, probably more clarification and explanation is welcome.

Are sufficient details of methods and materials provided to allow replication by others?

Partly

Is the rationale for creating the dataset(s) clearly described?

Yes

Are the datasets clearly presented in a useable and accessible format?

Yes

Are the protocols appropriate and is the work technically sound?

Yes

Reviewer Expertise:

Taxonomy, Karyology, Biodiversity Information

I confirm that I have read this submission and believe that I have an appropriate level of expertise to confirm that it is of an acceptable scientific standard.

# Reviewer report: Reviewer response for version 1

**DOI:** 10.21956/wellcomeopenres.29322.r156291

**Published:** 2026-07-09

**Authors:** Jose Martinez

**Affiliations:** 1 University of Florida, Gainesville, Florida, USA

*Orthosia cerasi* (Fabricius, 1775), the Common Quaker, has now been characterized through a high-quality chromosome-level genome assembly presented by Lees and Crumpton. The assembly comprises 99.88% of the genome scaffolded into 29 chromosomal pseudomolecules, including a fully resolved Z sex chromosome. In addition, the complete mitochondrial genome has been assembled, and gene annotation identified 15,702 protein-coding genes. The careful application of established sequencing, assembly, and annotation methodologies has produced a genomic resource of exceptional quality that will serve as an important reference for future studies of Noctuidae and Lepidoptera.

 This resource is particularly valuable because of its comprehensive documentation and standardized presentation. The rationale for generating the genome is clearly articulated, and the sequencing, assembly, and annotation workflows are described in a transparent and reproducible manner. The chromosome-level assembly, together with extensive gene annotation, supporting illustrations, and its integration into the Darwin Tree of Life, ensures that the dataset is readily accessible and broadly applicable to comparative, ecological, and functional genomic research. 

 Beyond its intrinsic scientific value, the genome of O. cerasi provides an important resource for understanding the biology of one of the most widespread and recognizable Palearctic noctuid moths. The Common Quaker is a widespread, univoltine species inhabiting woodlands, suburban gardens, and other temperate habitats across Europe and western Asia, where its larvae feed on a wide range of broad-leaved trees. Recent studies indicate that populations in the United Kingdom are declining, with these reductions associated with rising temperatures, phenological mismatches between larval development and host-plant availability, and sensitivity to both temperature and artificial light. Consequently, this genome provides a valuable foundation for investigating the genetic basis of climate responses, developmental biology, phenology, host-plant interactions, and population dynamics. By establishing a high-quality genomic reference for this ecologically important species, this dataset represents a significant contribution to entomology, genomics, conservation biology, and our broader understanding of the biological consequences of environmental change.

Are sufficient details of methods and materials provided to allow replication by others?

Yes

Is the rationale for creating the dataset(s) clearly described?

Yes

Are the datasets clearly presented in a useable and accessible format?

Yes

Are the protocols appropriate and is the work technically sound?

Yes

Reviewer Expertise:

Evolutionary Biology, Ecology, IPM, Integrative Biology, Entomology, Genomics

I confirm that I have read this submission and believe that I have an appropriate level of expertise to confirm that it is of an acceptable scientific standard.
